# Toward Realistic Autonomous Driving Dataset Augmentation: A Real–Virtual Fusion Approach with Inconsistency Mitigation

**DOI:** 10.3390/s26030987

**Published:** 2026-02-03

**Authors:** Sukwoo Jung, Myeongseop Kim, Jean Oh, Jonghwa Kim, Kyung-Taek Lee

**Affiliations:** 1 Contents Convergence Research Center, Korea Electronics Technology Institute, Seongnam-si 13449, Republic of Korea; myeongseopkim@keti.re.kr (M.K.); ktechlee@keti.re.kr (K.-T.L.); 2 Robotics Institute, Carnegie Mellon University, Pittsburgh, PA 15213, USA; hyaejino@andrew.cmu.edu; 3 MORAI Inc., Seoul 06168, Republic of Korea

**Keywords:** autonomous driving, dataset augmentation, real-virtual fusion, inconsistency mitigation, object recognition

## Abstract

Autonomous driving systems rely on vast and diverse datasets for robust object recognition. However, acquiring real-world data, especially for rare and hazardous scenarios, is prohibitively expensive and risky. While purely synthetic data offers flexibility, it often suffers from a significant reality gap due to discrepancies in visual fidelity and physics. To address these challenges, this paper proposes a novel real–virtual fusion framework for efficiently generating highly realistic augmented image datasets for autonomous driving. Our methodology leverages real-world driving data from South Korea’s K-City, synchronizing it with a digital twin environment in Morai Sim (v24.R2) through a robust look-up table and fine-tuned localization approach. We then seamlessly inject diverse virtual objects (e.g., pedestrians, vehicles, traffic lights) into real image backgrounds. A critical contribution is our focus on inconsistency mitigation, employing advanced techniques such as illumination matching during virtual object injection to minimize visual discrepancies. We evaluate the proposed approach through experiments. Our results show that this real–virtual fusion strategy significantly bridges the reality gap, providing a cost-effective and safe solution for enriching autonomous driving datasets and improving the generalization capabilities of perception models.

## 1. Introduction

The rapid advancement of autonomous driving technology promises innovative changes in transportation, enhancing safety, efficiency, and accessibility [1]. A cornerstone of reliable autonomous driving systems lies in their robust perception capabilities, particularly accurate and real-time object recognition from various sensors such as cameras, LiDAR, and IMUs [2,3,4,5]. Deep learning models, exemplified by architectures like YOLOv7 [6], YOLOv8 [7], YOLOv11 [8], and YOLOE [9], have shown remarkable success in object detection tasks, but their performance is critically dependent on the availability of vast, diverse, and high-quality training datasets [10].

However, the acquisition of such extensive datasets from real-world scenarios presents significant challenges. Collecting real data, especially for rare, extreme, or hazardous events (e.g., unexpected pedestrian behavior, complex traffic interactions at high speeds), is prohibitively expensive, time-consuming, and carries inherent safety risks [11,12,13,14,15]. These corner cases are precisely where autonomous driving systems need the most robust training and validation to ensure safety and reliability. Addressing this long-tail distribution—where critical scenarios appear infrequently—is essential for robust perception systems. Recent studies have highlighted data augmentation as a key strategy to resolve such class imbalance problems [16,17]. Consequently, researchers have increasingly turned to synthetic data generated from virtual environments, leveraging advanced simulators and digital twins [18,19,20,21,22,23,24,25,26].

Purely synthetic data, while offering unparalleled control over scenario generation and automatic ground truth labeling, often suffers from a significant reality gap. This discrepancy arises from differences in visual fidelity, material properties, lighting conditions, and sensor noise characteristics between simulated and real environments. Models trained solely on synthetic data frequently exhibit degraded performance when deployed in the real world, hindering their generalization capabilities. Efforts to bridge this gap often involve sophisticated domain adaptation techniques [10], but a more direct approach that maximizes the realism of synthetic data generation remains crucial.

To address these limitations, this paper proposes a novel framework for generating highly realistic augmented image datasets by fusing real-world backgrounds with injected virtual objects. Our core idea is to leverage the authentic visual context of real driving scenes while injecting synthetic elements that represent challenging or rare scenarios. Specifically, we utilize real driving data from South Korea’s K-City, a renowned autonomous driving test facility, and a sophisticated digital twin environment created in the Morai Sim simulator [27,28]. This fusion approach aims to combine the best of both worlds: the high fidelity of real backgrounds with the flexibility and safety of virtual object generation. Previous work by Jung et al. has demonstrated strong capabilities in moving object detection and 3D reconstruction using various sensor modalities and deep learning [2,3,4], forming a foundation for robust sensor data processing in real environments.

A critical aspect of our framework is the mitigation of inconsistencies that inevitably arise when superimposing virtual objects onto real images. Simple cut-and-paste methods often result in visual artifacts (e.g., unrealistic lighting, improper boundaries), which can mislead perception models. Our approach integrates several advanced techniques, including accurate real-to-virtual synchronization of camera poses and illumination estimation from real scenes to drive physically based rendering of virtual objects. Furthermore, we employ color transfer techniques [29] to harmonize the visual appearance of injected objects with the background. This meticulous attention to detail ensures that the injected virtual objects appear as naturally integrated as possible, thereby minimizing the reality gap for the generated augmented datasets.

The primary contributions of this paper are summarized as follows:

- We propose an efficient and novel real–virtual fusion framework that leverages real-world driving data from K-City and a digital twin in Morai Sim for autonomous driving dataset augmentation.
- We develop a robust real–virtual synchronization method, combining a look-up table for initial alignment with a fine-tuned localization algorithm, to accurately map real camera poses to the virtual environment.
- We implement an advanced virtual object injection pipeline focusing on inconsistency mitigation, incorporating illumination matching and color transfer to enhance the photorealism of augmented images.
- We demonstrate the effectiveness of our framework through two comprehensive experiments: quantitatively assessing the perceptual realism of the generated augmented images using a pre-trained YOLOv7 model, and evaluating the improved object recognition performance and robustness of a fine-tuned YOLOv7 model on a real-world dataset (BDD100k [30]).

The remainder of this paper is structured as follows. Section 2 reviews related work in autonomous driving datasets, simulation, and data augmentation. Section 3 details our proposed real–virtual fusion methodology. Section 4 presents the experimental setup, results, and qualitative analysis. Finally, Section 5 concludes the paper and outlines future work.

## 2. Related Works

This section reviews existing literature on autonomous driving datasets, simulation technologies, data augmentation strategies, and techniques for bridging the reality gap between synthetic and real-world data.

### 2.1. Autonomous Driving Datasets and Perception

High-quality and diverse datasets are essential for training and validating perception models in autonomous driving. Traditional datasets mainly consist of annotated real-world data, capturing various environmental conditions and traffic scenarios. Several studies have focused on improving object detection using camera and IMU sensors [2,3,4]. For instance, Jung et al. applied Mask R-CNN for instance segmentation to enhance detection performance [3]. Furthermore, multi-sensor fusion, such as combining LiDAR and camera data, has proven effective for robust 3D object detection, particularly in complex traffic environments [5,31]. In the field of 3D reconstruction, sensor fusion techniques like ToF-stereo fusion have been explored to generate detailed environmental maps [32].

### 2.2. Autonomous Driving Simulation and Digital Twins

Real-world data collection is costly, time-consuming, and often hazardous. Consequently, autonomous driving simulation has become a critical tool for development and testing [13,14,21]. Modern simulators provide customized environments and can generate diverse scenarios, including corner cases that are difficult to encounter in reality. Digital Twin technology, which creates virtual replicas of physical systems, plays a key role in these simulations [1,19,20]. These frameworks enable high-fidelity simulations and proactive safety validation [1,18]. Accurately modeling virtual sensors to mimic real-world outputs is crucial for testing perception algorithms’ reliability [19]. Additionally, Mixed Reality (MR) integrates virtual elements into real-world driving, allowing specific scenarios to be tested with real vehicles [12,25,26]. However, these approaches primarily focus on the system architecture for Hardware-in-the-Loop (HIL) or Vehicle-in-the-Loop (VIL) verification to test algorithms in real time. This evolution of simulation platforms aims to create more realistic and comprehensive virtual testing environments [15,21].

### 2.3. Data Augmentation and Reality Gap Mitigation

While synthetic data offers significant advantages, the reality gap between synthetic and real data remains a primary challenge. This gap often leads to poor generalization of models trained on synthetic data when applied to the real world [10]. Various strategies have been proposed to address this issue:

- Domain Adaptation: Techniques such as unsupervised domain adaptation reduce the discrepancy between source (synthetic) and target (real) domains. This enables models to perform well on real data without requiring extensive real-world annotations [10].
- Generative Models: Recent advances in generative AI, such as Neural Radiance Fields (NeRF), offer promising methods for generating high-fidelity synthetic data [23,24]. Generative AI-based simulations are also emerging to create immersive mixed-reality environments for vehicle testing [22].
- Rendering-Based Augmentation: Injecting synthetic objects into real-world scenes is a powerful data augmentation strategy. For example, LiDAR-Aug [33] composites virtual objects into real LiDAR scans for 3D detection. While effective for geometric data, this approach does not address the photometric inconsistencies inherent in camera sensors. Similar principles apply to camera data, but maintaining visual consistency is critical. Simple cut-and-paste methods often introduce artifacts due to mismatched lighting, shadows, or occlusions. Color transfer techniques, such as those by Reinhard et al. [29], provide a baseline for matching visual properties between different images. However, robust augmentation requires addressing both geometric and photometric inconsistencies to achieve seamless real–virtual fusion.

Building upon these works, our research focuses on a robust real–virtual fusion framework. Unlike prior works that often rely on simple overlays or geometric-only fusion, we aim to generate augmented datasets with high visual realism by minimizing the domain gap through our automated inconsistency mitigation pipeline. This effectively enhances the performance of autonomous driving perception models.

## 3. Proposed Methodology

**Figure 1:**
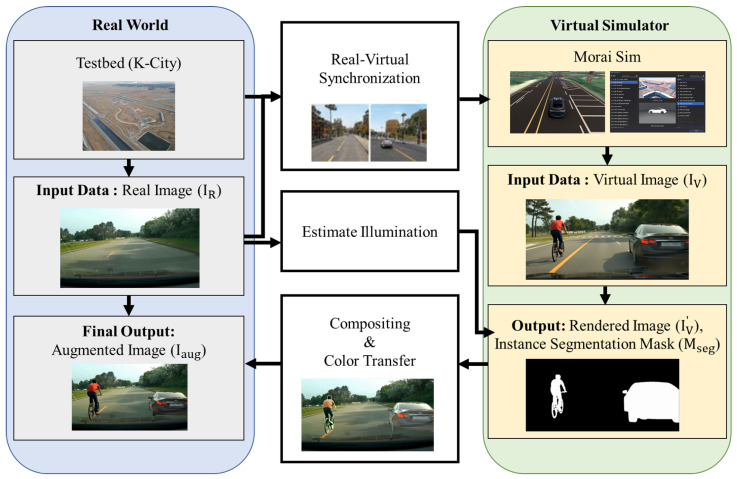
The overall pipeline of the proposed virtual object injection and augmented image generation method.

This section details our proposed real–virtual fusion framework for generating highly realistic augmented image datasets. We aim to overcome the limitations of purely synthetic or real-world data by leveraging the authenticity of real backgrounds and the flexibility of virtual object generation. An overview of the entire pipeline is illustrated in Figure 1.

### 3.1. Real–Virtual Synchronization

Accurate synchronization between the real-world driving environment (K-City) and its digital twin in Morai Sim is fundamental for seamlessly injecting virtual objects. This process ensures that the virtual camera’s pose within the simulator precisely matches the real camera’s pose when the background image was captured. Given that real-time synchronization is not a strict requirement for offline dataset generation, our approach focuses on achieving high precision in pose estimation and alignment.

#### 3.1.1. Initial Alignment via Look-Up Table

The first step involves establishing an initial correspondence between the global coordinate systems of K-City and the Morai Sim digital twin. We capture image and Inertial Measurement Unit (IMU) data using a ZED 2 stereo camera (which includes IMU functionality) in the K-City testbed. The Morai Sim digital twin of K-City is constructed with precise geographical information, including High-Definition (HD) maps. We create a Look-up Table (LUT) that maps specific real-world positions and vehicle orientations from the K-City environment to their corresponding locations and poses within the Morai Sim’s virtual map. This LUT serves as a coarse initial alignment. For a given real image captured at a specific timestamp *t*, with an associated global position and vehicle orientation, we query the LUT to retrieve the closest corresponding virtual pose $P_{V}^{\mathrm{init}}\left(t\right)=\left(X_{V},Y_{V},Z_{V},{\mathrm{roll}}_{V},{\mathrm{pitch}}_{V},{\mathrm{yaw}}_{V}\right)$ in the Morai Sim environment. This provides a robust starting point for subsequent fine-tuning.

#### 3.1.2. Fine-Tuned Localization Using Visual–Inertial Odometry

While the Look-up Table provides a good initial guess, minor discrepancies (e.g., due to GPS drift, map inaccuracies, or slight misalignments in digital twin construction) can exist. To achieve highly accurate camera pose synchronization, we employ a fine-tuned localization algorithm based on Simultaneous Localization and Mapping (SLAM) principles. From the real camera images, we extract visual features (ORB features) and track them across consecutive frames to estimate relative camera motion. Concurrently, the IMU data provides high-frequency angular velocity and linear acceleration measurements. These inputs are fused to obtain Visual–Inertial Odometry (VIO) based on the algorithm proposed in our previous work [34]. The system is implemented in-house using C++ and the OpenCV 4.10.0 library. By combining the strengths of visual odometry and inertial odometry, we estimate the precise relative pose changes of the real vehicle. These relative pose changes are then used to refine the initial pose obtained from the Look-up Table. Specifically, for each real camera image sequence, we perform a local optimization of the camera trajectory, ensuring consistency between the visual features and IMU measurements. This process effectively corrects for short-term drifts and significantly improves the local accuracy of the real camera’s pose. The refined real camera pose $P_{R}\left(t\right)=\left(X_{R},Y_{R},Z_{R},{\mathrm{roll}}_{R},{\mathrm{pitch}}_{R},{\mathrm{yaw}}_{R}\right)$ is then used to set the virtual camera’s pose $P_{V}\left(t\right)$ within Morai Sim, aiming for $P_{V}\left(t\right)=P_{R}\left(t\right)$.

The cumulative refined real camera pose $P_{R}\left(t_{k}\right)$ at timestamp $t_{k}$ can be expressed as a composition of the initial pose and the subsequent relative transformations:

(1) $$P_{R}\left(t_{k}\right)=P_{V}^{\mathrm{init}}\left(t_{0}\right)\circ \left(\prod_{i=0}^{k-1}\Delta T_{t_{i},t_{i+1}}^{\mathrm{VIO}}\right)$$

where $P_{V}^{\mathrm{init}}\left(t_{0}\right)$ is the initial pose obtained from the Look-up Table at the starting timestamp $t_{0}$, $\Delta T_{t_{i},t_{i+1}}^{\mathrm{VIO}}$ represents the estimated relative pose transformation between consecutive timestamps $t_{i}$ and $t_{i+1}$, and ∘ denotes the composition operation of rigid body transformations.

#### 3.1.3. Drift Correction and Global Consistency

**Figure 2:**
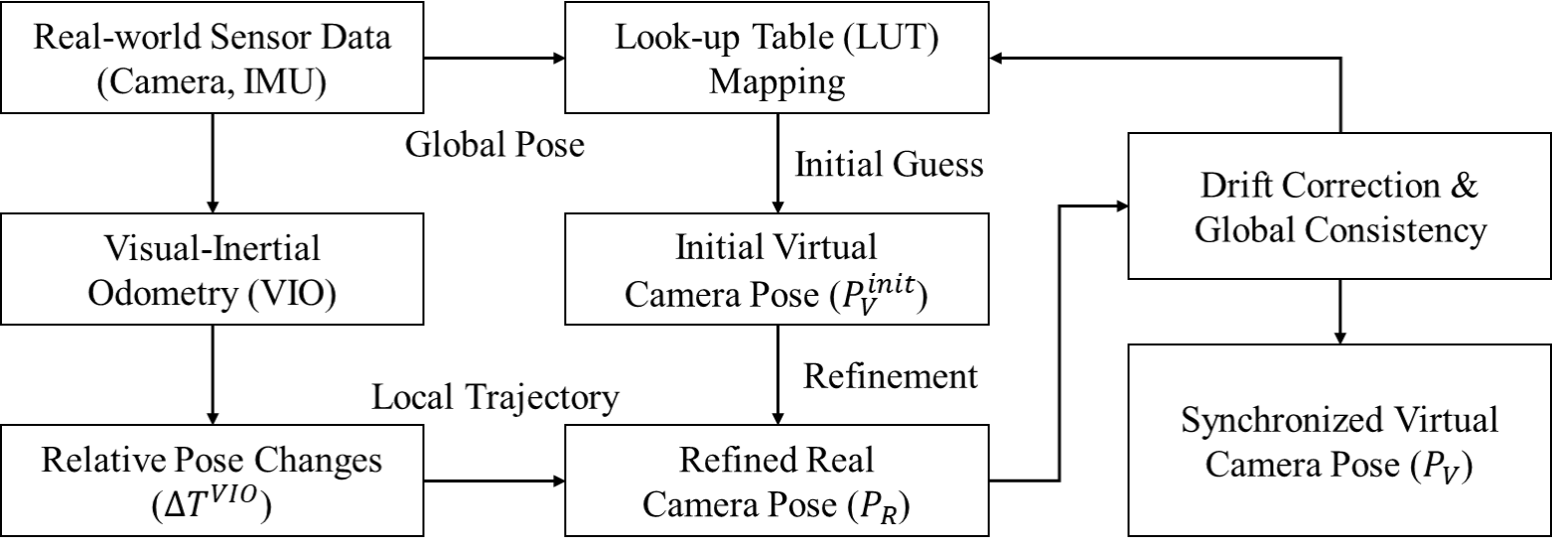
Conceptual Diagram of Real–Virtual Synchronization Framework.

Long-term VIO or SLAM-based systems are prone to cumulative drift. To prevent this, our approach incorporates periodic drift correction to maintain global consistency. At regular intervals, the locally refined VIO poses are re-referenced against the LUT’s global initial alignments. This acts as a loop closure mechanism, effectively re-anchoring the local trajectory to a globally consistent frame and distributing any accumulated drift across the trajectory segment. This ensures that the virtual camera’s trajectory remains globally consistent with the real vehicle’s path, even over extended periods of data acquisition, minimizing discrepancies for subsequent virtual object injection. Figure 2 illustrates the overall flowchart of this real–virtual synchronization framework.

### 3.2. Virtual Object Injection and Augmented Image Generation

With accurate real–virtual synchronization, the next step is to realistically inject virtual objects into the real background images. This stage focuses on minimizing visual and geometric inconsistencies, a key challenge in mixed-reality data generation.

#### 3.2.1. Virtual Object Placement and Scene Composition

Once the real camera’s pose $P_{R}\left(t\right)$ is precisely transferred to the virtual camera $P_{V}\left(t\right)$ in Morai Sim, virtual objects from our library (e.g., vehicles, pedestrians, traffic signs) are dynamically placed into the virtual scene. In this study, we intentionally utilized real background frames captured in K-City that are relatively free of dynamic foreground objects. This design choice provides a blank canvas, allowing for the unrestricted placement of virtual agents without the immediate need for complex occlusion handling between real and virtual dynamic objects. Leveraging this flexibility, virtual objects are strategically positioned to create diverse and challenging scenarios, including rare events and dangerous interactions, effectively enabling the infinite generation of training data. The Morai Sim environment, as a digital twin of K-City, ensures that this virtual object placement respects the underlying road network and physical constraints.

#### 3.2.2. Illumination Estimation and Color Transfer

A major source of visual inconsistency is mismatched lighting between real backgrounds and virtual objects. To address this, we perform illumination estimation from the real background image $I_{R}$. Simplified statistical models are employed to approximate the real-world lighting environment. The estimated illumination parameters are then used to adjust the virtual image rendered by Morai Sim. The final augmented image $I_{aug}$ is generated by compositing the realistically rendered virtual objects onto the real background image. Since the chosen real backgrounds are free of foreground objects, the compositing process involves directly superimposing the virtual object pixels. Morai Sim provides a precise segmentation mask ($M_{seg}$) for each rendered virtual object. The augmented image $I_{aug}$ is formed by replacing the corresponding pixels in the real background $I_{R}$ with the rendered virtual object pixels $I_{V}$ according to $M_{seg}$. It is important to note that $M_{seg}$ corresponds strictly to the object’s geometry, excluding cast shadows. While shadows contribute to visual realism, including them in the mask implies that the generated bounding boxes would essentially encompass the shadows, leading to inaccurate ground truth labels for detection tasks. Therefore, we prioritized label precision by limiting the compositing region to the object itself. As a final harmonization step, we apply a color transfer technique [29] between the source (rendered virtual object region $I_{V}$) and target (corresponding real background region $I_{R}$) within the object’s mask. The compositing process for each pixel coordinate *p* can be formally defined as:

(2) $$I_{aug}\left(p\right)=M_{seg}\left(p\right)\cdot I_{V}^{\prime }\left(p\right)+\left(1-M_{seg}\left(p\right)\right)\cdot I_{R}\left(p\right)$$

where $I_{R}$ is the real background image, $I_{V}^{\prime }$ is the rendered virtual object image after color transfer, and $M_{seg}\left(p\right)$ is a binary mask value which is 1 if pixel *p* belongs to the object geometry, and 0 otherwise.

### 3.3. Automated Ground Truth Labeling

A significant advantage of generating data from a virtual environment is the automated provision of precise, rich ground truth labels. For each injected virtual object, Morai Sim automatically generates a comprehensive set of labels, including 2D bounding box coordinates, instance segmentation masks, and class labels. These rich, accurate, and consistent labels are immediately available for training and evaluating perception models, eliminating the time-consuming and error-prone manual annotation process required for real-world data.

## 4. Experiments

To validate the effectiveness and realism of our proposed real–virtual fusion framework, we conducted a series of comprehensive experiments. This section details the experimental setup, the datasets generated, the evaluation metrics, and the results obtained from our experiments.

### 4.1. Experimental Setup

#### 4.1.1. Datasets

Our experiments are based on a combination of real-world data, a digital twin simulator, and the augmented datasets generated by our framework.

- Real-World Data (K-City): We collected several hours of driving data from the K-City testbed in Hwaseong, South Korea. This data includes high-resolution camera images (1920 × 1080, 30 fps) and time-synchronized IMU measurements, which are crucial for our real-virtual synchronization process described in Section 3.1.
- Virtual Environment (Morai Sim): A high-fidelity digital twin of the K-City environment was utilized, built within the Morai Sim simulator. This virtual environment provides virtual sensor data corresponding to real-world coordinates.
- Generated Datasets: We generated two distinct datasets for our experiments, both based on the 7 target classes (Person, Bicycle, Car, Motorcycle, Bus, Truck, and Traffic Light).
  - $D_{\mathrm{aug}\_\mathrm{train}}$: A large-scale training dataset of 4229 augmented images. This set was created by injecting the 7 classes of virtual objects into the real K-City backgrounds, with approximately 500 images per class, covering various challenging scenarios (e.g., varying distances, angles, and potential near-collision trajectories). This dataset was generated in two versions: one with our full pipeline including color transfer, and one without, for ablation studies.
  - $D_{\mathrm{aug}\_\mathrm{test}}$: A test dataset of 553 augmented images (approx. 80 images per class). This set was created for Experiment 1.
- Real-World Test Set ($D_{\mathrm{real}\_\mathrm{test}}$): We utilize the official BDD100k [30] object detection validation split for Experiment 2. This large-scale dataset, comprising 10,000 images, provides diverse, real-world driving scenarios and includes annotations for all 7 of our target classes, enabling a robust evaluation of real-world generalization performance.

#### 4.1.2. Implementation Details

We employ YOLOv7 [6] as our primary baseline model due to its established performance in real-time autonomous driving scenarios and its representative anchor-based architecture. To further validate the generalization capability of our augmented data across different detector types, we also conduct experiments using YOLOv8 (a representative anchor-free model) and the recently released YOLOv11. All models were initialized with weights pre-trained on the MS COCO dataset. This model (YOLOv7_base) serves as our baseline for both Experiments 1 and 2. We fine-tuned the YOLOv7_base model on our $D_{\mathrm{aug}\_\mathrm{train}}$ dataset for 100 epochs with an initial learning rate of 0.001 and a batch size of 16. We utilized the Stochastic Gradient Descent (SGD) optimizer with a momentum of 0.937 and a weight decay of 0.0005. Additionally, a linear warm-up strategy was employed for the first 3 epochs to ensure stable convergence. We trained two variants to assess the impact of our inconsistency mitigation pipeline: YOLOv7_aug (fine-tuned on augmented data without color transfer) and YOLOv7_ours (fine-tuned on augmented data with color transfer [29], using the proposed method). All training and testing were conducted on a workstation equipped with an NVIDIA GeForce RTX 3090Ti GPU and 128 GB of RAM.

#### 4.1.3. Evaluation Metrics

To provide a comprehensive evaluation, we adopt the standard metrics used in object detection.

- **Precision (P):** The accuracy of positive predictions. It is defined as:
  (3) $$P=\frac{TP}{TP+FP}$$
  where TP is True Positives and FP is False Positives.
- **Recall (R):** The ability of the model to find all relevant ground truths. It is defined as:
  (4) $$R=\frac{TP}{TP+FN}$$
  where FN is False Negatives.
- **F1-Score:** The harmonic mean of Precision and Recall, providing a single score that balances both metrics.
  (5) $$F1=2\cdot \frac{P\cdot R}{P+R}$$
- **mAP@0.5:** The mean Average Precision (mAP) calculated at a fixed Intersection over Union (IoU) threshold of 0.5. AP for a single class is the area under the Precision-Recall curve:
  (6) $$AP=\int_{0}^{1}p\left(r\right)\;dr$$
  mAP is the average of AP scores across all *C* classes (in our case, C=7).
  (7) $$mAP=\frac{1}{C}\sum_{i=1}^{C}AP_{i}$$

### 4.2. Experiments and Results

We conducted two main experiments to thoroughly evaluate our proposed real-virtual fusion framework.

#### 4.2.1. Experiment 1: Perceptual Realism Test

This experiment aims to quantitatively assess the perceptual fidelity of our generated augmented data. The hypothesis is that if our synthetic objects are perceptually realistic, an off-the-shelf object detection model (YOLOv7_base) that has never seen our synthetic data should still be able to detect them with high accuracy. This provides a strong quantitative indicator of how real our augmented objects appear to a model trained exclusively on real data. We evaluated the performance of the YOLOv7_base model on our $D_{\mathrm{aug}\_\mathrm{test}}$ dataset, which contains the 7 target object classes injected into real K-City backgrounds. Table 1 shows the detection results for each class.

The high mAP@0.5 scores across all classes indicate that the YOLOv7_base model successfully recognized the injected virtual objects despite having no prior exposure to our synthetic data. Considering that the standard YOLOv7 achieves approximately 69.7% mAP@0.5 on the MS COCO validation set [6], our result of 80.7% suggests a remarkably high level of realism in our augmented data. This provides strong evidence that our framework effectively bridges the reality gap of virtual object injection.

To further validate the perceptual realism without architectural bias, we additionally employed the YOLOE detector in a prompt-free zero-shot mode. Unlike YOLOv7, this model was used to infer the augmented images without any prior knowledge of our specific dataset. As presented in Table 2, the model successfully detected a significant number of injected objects.

Notably, classes with complex textures such as Person, Bus, and Truck achieved high average confidence scores (above 0.83), indicating effective texture harmonization. While statistical metrics like FID or KID could provide complementary insights regarding distribution distances, these task-based evaluation results strongly confirm that our proposed pipeline generates synthetic data with sufficient objectness and realism to be utilized by state-of-the-art detectors.

#### 4.2.2. Experiment 2: Performance Improvement Test

This experiment evaluates the effectiveness of training object detection models with our augmented data to improve performance on a real-world dataset. We fine-tuned the baseline models on our $D_{\mathrm{aug}\_\mathrm{train}}$ dataset and evaluated them on the BDD100k validation split ($D_{\mathrm{real}\_\mathrm{test}}$).

To demonstrate the universality of our approach, we extended the evaluation beyond YOLOv7 to include YOLOv8 and YOLOv11. For each architecture, we compared the pre-trained baseline (Base) against the model fine-tuned on our augmented dataset (Ours). The comparative results across all architectures are presented in Table 3.

The results in Table 3 demonstrate that training with our augmented data significantly improves object detection performance on the real-world BDD100k dataset. The YOLOv7_ours model achieved an overall mAP@0.5 of 56.4%, outperforming the baseline YOLOv7_base (45.7%) by 10.7%.

Furthermore, we conducted an ablation study to isolate the contribution of our inconsistency mitigation technique. Table 4 compares the performance of YOLOv7_aug (trained on data without color transfer) and YOLOv7_ours (trained on data with color transfer).

The results reveal the critical role of our color transfer technique. YOLOv7_ours consistently achieved higher mAP scores across all classes compared to YOLOv7_aug, with an overall improvement of +5.9%. This indicates that simply overlaying virtual objects is insufficient; harmonizing the visual properties (e.g., color tone, brightness) between virtual objects and real backgrounds is essential for maximizing the realism and utility of synthetic training data.

### 4.3. Qualitative Analysis

**Figure 3:**
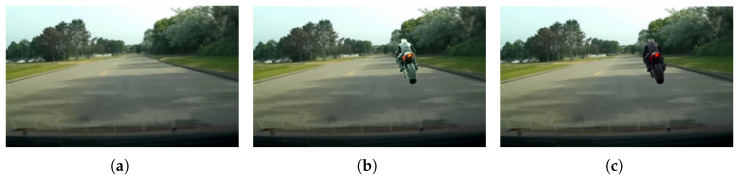
Qualitative comparison of our augmentation pipeline: (**a**) original background image under daylight condition. (**b**) naive virtual object injection without color transfer. (**c**) proposed method with color transfer, demonstrating seamless integration and high perceptual realism.

**Figure 4:**
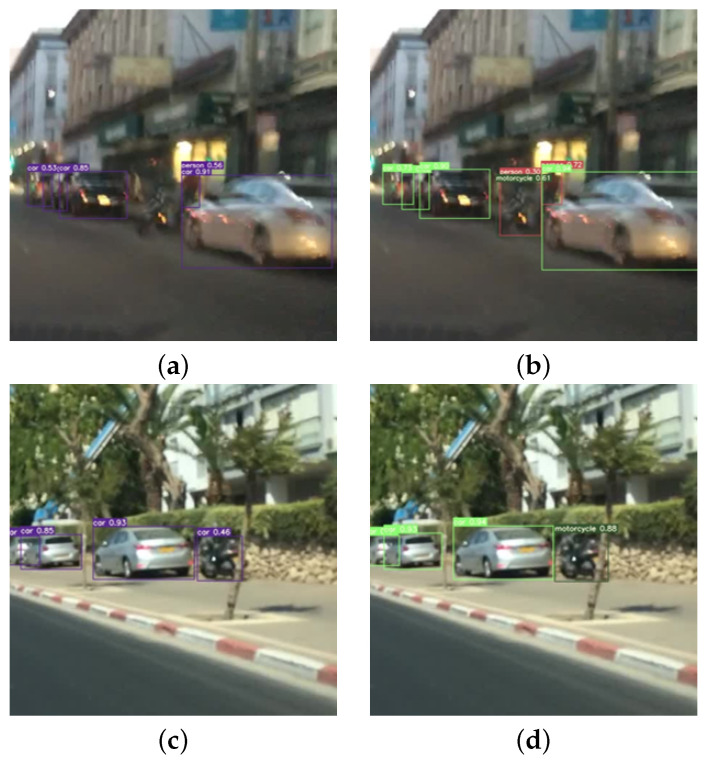
Visual comparison of detection performance on challenging BDD100k scenarios. (**a**,**c**) the baseline YOLOv7_base model misses critical objects such as Motorcycles due to complex backgrounds or low visibility. (**b**,**d**) our proposed YOLOv7_ours model correctly identifies and localizes these objects, validating the effectiveness of our real-virtual fusion augmentation in enhancing model sensitivity for rare classes.

In addition to quantitative metrics, we provide a qualitative analysis to visually demonstrate the quality of our augmented data and the improved detection performance. Figure 3 illustrates examples of our generated augmented images, showcasing the seamless integration of virtual objects into diverse real backgrounds. Figure 4 presents detection results on challenging BDD100k images, comparing the baseline model with our YOLOv7_ours model. These examples highlight instances where the baseline model failed to detect or misclassified objects that our augmented-data-trained model successfully identified.

## 5. Conclusions

This paper proposed a real–virtual fusion framework to generate realistic augmented datasets for autonomous driving. Our approach effectively addresses the data scarcity problem by injecting virtual objects into real-world backgrounds. Experimental results validated the effectiveness of the proposed method. First, the perceptual realism assessment showed an 80.7% mAP@0.5 on the augmented test set, confirming high visual fidelity. Second, fine-tuning the YOLOv7 model with our augmented dataset improved performance on the real-world BDD100k dataset by 10.7% mAP compared to the baseline. Notably, we observed significant performance gains in data-scarce classes such as ‘Motorcycle’ (+15.2%) and ‘Bicycle’ (+13.2%). The ablation study further confirmed that color transfer is a critical component for reducing the domain gap between real and virtual data.

The generation process currently relies on fixed real-world backgrounds, which limits the ability to simulate varying weather or lighting conditions without additional generative models. Consequently, we observed that while the relative performance gains were substantial, the absolute detection accuracy for classes heavily dependent on specific lighting conditions (e.g., traffic lights) remained lower than that of daytime-dominant classes. This suggests that while our pipeline is effective, increasing background diversity is essential for achieving broader generalization across all environmental conditions.

Scalability to an even larger diversity of virtual objects and complex dynamic scenarios remains an area for further exploration. Moreover, while our framework significantly improves detection, its impact on other perception tasks (e.g., segmentation, manufacturing sites) could be investigated. Furthermore, this study focused on the overall pipeline of injecting virtual objects into synchronized real-world coordinates. The specific impact of synchronization accuracy on training efficiency, and a comparative analysis between context-aware placement (via digital twin) and random placement strategies, remain open questions. Future work will explore these geometric aspects, along with adaptive color transfer methods and the integration of Generative Adversarial Networks (GANs) to further refine realism. Specifically, generating more diverse and challenging occlusion scenarios in synthetic environments—such as heavy traffic where objects are partially hidden—and developing robust methods to handle them is a critical issue for future research to further close the reality gap. 

## Figures and Tables

**Table 1:** Detection Performance of YOLOv7_base on ($D_{\mathrm{aug}\_\mathrm{test}}$).

Class	mAP@0.5	P	R	F1-Score
Person	78.5%	85.2%	75.1%	0.798
Car	91.2%	93.5%	90.1%	0.918
Bicycle	75.8%	81.0%	72.5%	0.765
Truck	83.1%	87.6%	80.0%	0.836
Bus	85.5%	89.1%	83.0%	0.859
Motorcycle	71.9%	77.2%	68.5%	0.726
Traffic Light	79.0%	84.5%	75.5%	0.798
Overall mAP@0.5	80.7%	-	-	-

**Table 2:** Quantitative evaluation of perceptual realism using YOLOE (Prompt-free mode).

Class	Detected Count	Avg. Confidence
Person	227	0.85
Bicycle	89	0.66
Car	498	0.56
Motorcycle	150	0.75
Bus	87	0.84
Truck	83	0.83
Traffic Light	373	0.54
Average	-	0.72

**Table 3:** Comparison of Detection Performance (mAP@0.5) Across Different Architectures on BDD100K Validation Set.

Class	YOLOv7		YOLOv8		YOLOv11		Avg. Gain
	**Base**	**Ours**	**Base**	**Ours**	**Base**	**Ours**	
Person	59.3	63.1	51.2	57.4	58.0	64.7	+5.6
Bicycle	38.0	50.8	33.7	51.1	40.4	49.8	+13.2
Car	66.2	69.5	56.2	70.5	66.2	70.7	+7.4
Motorcycle	37.3	51.9	38.6	55.9	38.5	52.2	+15.2
Bus	47.0	58.4	49.3	60.1	46.8	51.6	+9.0
Truck	40.1	51.2	54.6	59.2	40.7	52.9	+9.3
Traffic Light	32.2	39.9	26.6	48.9	28.9	49.6	+16.9
Overall mAP	45.7	56.4	44.3	57.5	45.6	55.9	+11.4

**Table 4:** Ablation Study: Effectiveness of Inconsistency Mitigation (Color Transfer) on YOLOv7.

Class	YOLOv7_aug			YOLOv7_ours			ΔmAP (Ours—Aug)
	**mAP**	**P**	**R**	**mAP**	**P**	**R**	
Person	60.5%	74.0%	54.2%	63.1%	78.5%	56.8%	+2.6%
Bicycle	44.2%	66.8%	40.5%	50.8%	72.4%	48.1%	+6.6%
Car	67.8%	80.5%	59.1%	69.5%	83.2%	62.4%	+1.7%
Motorcycle	45.6%	56.2%	48.3%	51.9%	65.7%	55.2%	+6.3%
Bus	52.1%	48.5%	55.4%	58.4%	62.0%	61.5%	+6.3%
Truck	45.3%	35.4%	60.2%	51.2%	48.6%	65.3%	+5.9%
Traffic Light	35.8%	58.2%	34.5%	39.9%	64.1%	38.2%	+4.1%
Overall	50.5%	-	-	56.4%	-	-	+5.9%

## Data Availability

The data generated and analyzed during the current study are not publicly available due to proprietary agreements with the K-City testbed and MORAI Inc. (developer of Morai Sim), but may be made available from the corresponding author upon reasonable request.
